# Switching up How You Get in Your Steps: Daily Activity Diversity and Cognitive Functioning

**DOI:** 10.1093/geroni/igab046.2222

**Published:** 2021-12-17

**Authors:** Colette Brown, Sangha Jeon, Yee To Ng, Soomi Lee, Susan Charles, Karen Fingerman

**Affiliations:** 1 University of California, Irvine, Irvine, California, United States; 2 University of Texas at Austin, Austin, Texas, United States; 3 University of South Florida, Tampa, Florida, United States

## Abstract

Active lifestyles are related to better cognitive health. More work is needed, however, to examine whether participating in a variety of daily activities (i.e., activity diversity) has unique importance beyond amount of activity. The current study examined associations between daily activity diversity and cognitive functioning among community-dwelling older adults (N = 313, ages 65-90). Participants completed a cognitive battery, then responded to ecological momentary assessments of their participation in 10 common activity types (e.g., exercise, chores, social visits, volunteering) every 3 hours for 5-6 days, and wore accelerometers to track daily step counts and duration of activity. Multiple regression models revealed that greater daily activity diversity related to higher overall cognitive functioning, executive functioning, memory, and crystallized intelligence. These associations remained significant after adjusting for step count and duration of activity. Findings suggest daily activity diversity has unique importance beyond sheer amount of activity for cognitive health in later adulthood.

